# Temporal and Spatial Analysis of Trace Metal Ecotoxicity in Sediments of Chaohu Lake, China

**DOI:** 10.3390/toxics12120923

**Published:** 2024-12-20

**Authors:** Wenguang Luo, Zongjun Li, Ran Yi, Lijuan Han, Senlin Zhu

**Affiliations:** 1 State Key Laboratory of Water Resources Engineering and Management, Wuhan University, Wuhan 430072, China; 2 Jingjiang Bureau of Hydrology and Water Resources Survey, Changjiang Water Resources Commission, Jingzhou 434000, China; jjlizongjun@cjh.com.cn; 3 Institute of Hydroecology, Ministry of Water Resources and Chinese Academy of Sciences, Wuhan 430079, China; qqyiranhh@163.com; 4 College of Hydro Science and Engineering, Taiyuan University of Technology, Taiyuan 030024, China; hanlijuan@tyut.edu.cn; 5 College of Hydraulic Science and Engineering, Yangzhou University, Yangzhou 225009, China

**Keywords:** trace metals, sediment re-suspension, release, species sensitivity, ecotoxicological risk

## Abstract

The species sensitivity distribution (SSD) analysis for aquatic ecosystems has been increasingly used in risk assessment. However, existing analyses of the impact of trace metals in lake sediments on aquatic organisms often neglect the spatiotemporal variability of trace metal release. This oversight can result in ecological risk assessments that lack specificity. To address this gap, we collected 32 core sediment samples from Lake Chaohu to systematically investigate the ecological toxicological risks posed by the release of eight trace metal indicators into the overlying water column under four hydrological scenarios throughout the year. Results indicated that only Cu, Pb, and Zn exhibit persistent toxicological risks. The comprehensive ecological toxicological risk of sediment trace metals showed spatial differences, increasing from the western region to the eastern region, i.e., western region < central region < eastern region. Seasonally, the risk levels are ordered as follows: May < September < November to April of the following year < June to August. The eastern region in summer (June to August) was identified as the high-risk area and period for trace metal pollution in sediments. Based on these conclusions, it is recommended to implement pollution control and environmental monitoring measures in the eastern region during the summer to effectively control the pollution and ecological risks of trace metals.

## 1. Introduction

Trace metals are pollutants known for their strong toxicity and non-degradability. Upon entering lakes with flow, a significant portion of trace metals accumulates in lake sediments [1,2,3]. However, due to exchange processes between sediment and overlying water, trace metals continuously leach into lake water through hydrodynamic processes, deteriorating water quality. Additionally, they can amplify through the food chain, posing severe threats to aquatic ecosystems and human health [4,5]. Aquatic organisms, integral parts of lake ecosystems, are particularly vulnerable to the toxic threat posed by trace metal pollutants in lake sediment. Consequently, the ecological risk assessment of trace metals in lake sediments has been a significant research focus. Recent studies on lakes in China and the world indicate that the overall potential ecological risks of typical trace metals such as Pb, Ni, Zn, Cu, Cd, and Hg have reached moderate levels [6,7]. However, the sensitivity of aquatic organisms to trace metals across different lakes among aquatic organisms across different ecosystems is not well understood.

Species sensitivity distribution (SSD), proposed by the US EPA in 1978 for establishing water quality criteria, has been widely applied in ecological risk assessment [8,9,10]. SSD describes the toxicity of a pollutant to a range of aquatic species by selecting a probability distribution and fitting an SSD curve, and it has been extensively used to assess the ecological risks of trace metals in lakes [11,12]. Compared to classical deterministic ecological risk assessment methods, SSD provides a more scientific approach by distributing the hazard levels of trace metals to different species from an ecosystem perspective, thus more accurately depicting the response of specific aquatic organisms to trace metal pollution [13,14].

Many studies have employed this method to assess the ecological risk of trace metal in lake sediments [15,16]. However, these studies have certain limitations. They often assessed the toxicity of trace metals to aquatic organisms solely based on their concentrations in sediments, without accounting for the doses released into the water column, which is an incomplete and potentially misleading approach. Studies have shown that disturbed sediment can release a large number of trace metals into the water upon resuspension [17,18]. However, under natural conditions, there are limitations to the depth of sediment disturbance, implying a maximum dose of trace metals that can enter the overlying water rather than an endless release [19]. Additionally, existing studies have paid less attention to the spatiotemporal heterogeneity of small-scale ecological risks. Lakes experience variations in water depth and sediment resuspension intensity over different periods [20], leading to dynamic changes in the total amount of trace metals transferred from sediment to overlying water. The risk to aquatic ecosystems posed by trace metals becomes significantly pronounced for shallow lakes in this regard.

Chaohu Lake, a large shallow lake in one of the most economically developed regions in China, the Yangtze River basin, has increased trace metal accumulation in sediments due to intensified human activities, leading to elevated concentrations and total amounts of trace metals in sediments [21,22]. The ecological risk of trace metals to the aquatic ecosystem, especially to aquatic organisms, has become crucial [23,24]. Its unique hydrological characteristics and ecological importance make it an ideal site to assess the environmental risks of heavy metal pollution. In this study, focusing on aquatic organisms, we applied the SSD method to assess the ecotoxicological risk of trace metals in surface sediments, using trace metal concentration data from sediment samples and toxicity data from the US EPA’s ECTOX database. We particularly investigated the spatial variation in the ecotoxicity risk of trace metals in sediments across different regions and seasons. Through this study, we aim to gain further insights into the overall trend of the ecological risk posed by trace metals in sediments to aquatic organisms in Lake Chaohu and other similar large shallow lakes.

## 2. Materials and Methods

### 2.1. Study Area

Chaohu Lake, one of the five major freshwater lakes in China, is situated in Hefei City (31.60° N, 117.87° E), Anhui Province, in the lower reaches of the Yangtze River system. The lake spans approximately 54.5 km from east to west, with an average width of 15.1 km from north to south, and it has a shoreline stretching 181 km. Covering a water surface area of around 780 km^2^, it holds a maximum volume of 48.10 billion m^3^, with an average water depth of 2.89 m (the minimum water depth is 0.29 m, and the maximum depth does not exceed 8 m). It is fed by a network of 35 rivers, including large ones such as the Hangbu River, Baishitian River, Pai River, Nanfei River, Tongyang River, Zhegao River, and Zhao River, which merge into the lake from the south, west, and north. The Yu Creek mouth in the east serves as its outlet to the Yangtze River.

**Figure 1:**
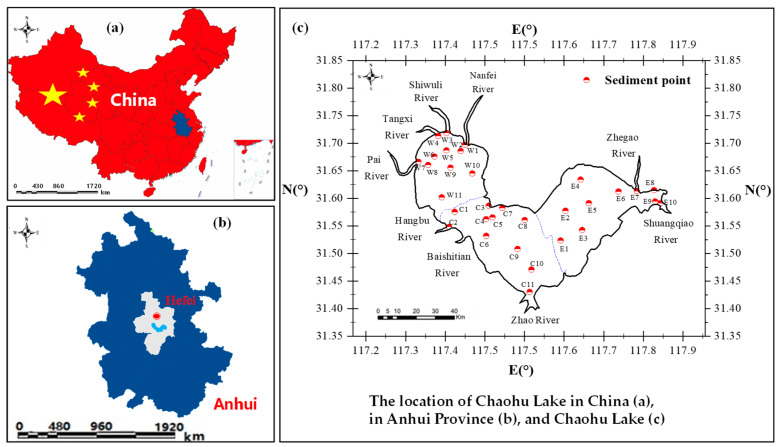
Sampling locations of the original sediment column cores of Lake Chaohu.

To provide a comprehensive representation of trace metal distribution across the lake, ensuring coverage of the major inflow areas and outlets, as well as capturing potential spatial variability in sediment contamination levels, 32 sediment core sampling sites were selected, evenly distributed across three regions, namely the western (denoted as “W”), central (denoted as “C”), and eastern (denoted as “E”) lake areas (see Figure 1).

### 2.2. Sediment Samples Collection and Detection

**Figure 2:**
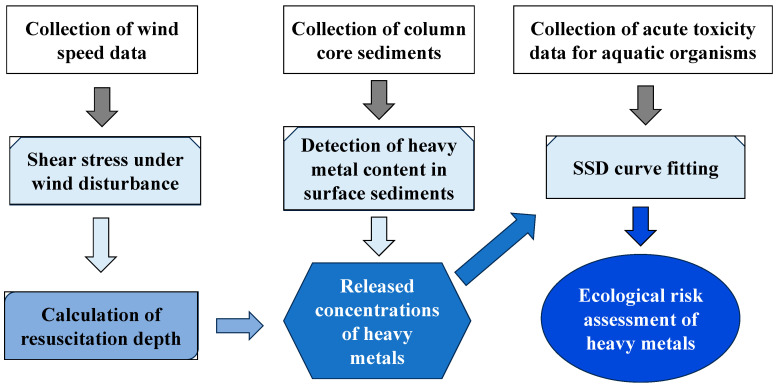
The main research process and method of this study.

The representative trace metal content of the sediment surface can be obtained by arranging 32 core sediment sampling sites in Lake Chaohu. The ecotoxicological risk of trace metals in sediments to aquatic organisms can be assessed by studying different sediment re-suspension depths to analyze the concentration of trace metals released into water bodies and combined with the most reasonable SSD curve selected. The specific research paths and methods are shown in Figure 2.

As can be seen from Figure 2, it is necessary to collect the trace metal content information of representative sediments at first. Sediment core samples were collected using the CORE-60 sampler (UWITEC, Austria) in the spring of 2019. To ensure representativeness, additional surface samples were collected from nearby locations to minimize channel influences. The current study focuses solely on the surface sediment samples, while the results of core analyses are not included here. The quantification of trace metals, including Cd, Hg, As, Pb, Cr, Cu, Zn, and Ni, was conducted following Chinese national standards. Cd and Pb were determined using graphite furnace atomic absorption spectrometry (GB/T 17141-1997). Hg and As were analyzed using the atomic fluorescence method (GB/T 22105.2-2008). Cr, Cu, and Zn were measured by flame atomic absorption spectrophotometry (HJ 491-2009, and GB/T 17138-1997), while Ni was quantified using flame atomic absorption spectrophotometry (GB/T 17139-1997). Quality control procedures included the use of reagent blanks, duplicates, and national standard reference materials (GBW07386, GBW07387, and GBW07388) [16]. The analytical results were consistent with the certified values, with recoveries ranging between 90% and 110%. Sample analysis was performed at the laboratory of Wuhan University.

Additionally, representative sediment cores (W9, C9, and E5) located very close to the central regions of the three areas of Lake Chaohu were selected for the analysis of sediment grain size, moisture content, and density along the vertical profile. The results showed that the median grain size of surface sediment (within 5 cm) was 6.76 μm, 11.83 μm, and 7.03 μm in the western, central, and eastern regions, respectively. The moisture content of surface sediment (within 5 cm) in the western, central, and eastern regions was 80.55%, 85.20%, and 80.05%, respectively. The density of surface sediment (within 5 cm) in the western, central, and eastern regions was 1.03 g/cm^3^, 1.03 g/cm^3^, and 1.05 g/cm^3^, respectively.

### 2.3. Construction of the SSD Curves

In this study, the toxicity hazard of trace metals to aquatic ecosystems was primarily analyzed through SSD curves constructed using laboratory toxicity data (USEPA, 2018). Acute toxicity data (half maximum effective concentration, EC50, or 50% lethal concentration, LC50) for algae, fish, crustaceans, and other invertebrates were retrieved from various public sources using data retrieval functions [25]. From the extensive toxicological dataset collected in this study, toxicity data representing the overall species toxicity of all aquatic organisms were selected as indicator indices.

Using the selected toxicity data, four distribution models—normal distribution, log-normal distribution, logistic distribution, and log-logistic distribution—were employed to calculate species mean acute values (SMAVs) for constructing SSD curves [26]. The most suitable SSD curve was determined based on the root mean square error (RMSE). Combining the results of the SSD curves with the concentrations of trace metals diffusing from sediment into the water column, the ecological risk posed by trace metals to lake aquatic organisms could be diagnosed.

### 2.4. Hydrological and Meteorological Data

Since the 1970s, Lake Chaohu has been regulated as an artificial reservoir, with its volume primarily controlled by human intervention. According to the current water level control method, as outlined in the “Interim Provisions on Flood Control and Drought Relief Scheduling for Lake Chaohu” (Provincial Flood Control Headquarters [2012] N.41), the water level is regulated at 8.00 m from June to August, 8.50 m in May and September, and controlled between 8.50 and 9.00 m during non-flood seasons [27]. Based on these four known water levels, the water surface area and volume of the lake can be further determined in this study, which is structured to investigate seasonal variations by analyzing data corresponding to each of these specific water level control periods.

Additionally, utilizing the daily average wind speed data from the Chaohu meteorological station in 2019, the distribution of monthly average wind speeds throughout the year was statistically analyzed [28]. Subsequently, representative monthly average wind speeds for each of the aforementioned four scenarios were selected based on the statistical results (Appendix A Table A1) for further analysis.

### 2.5. Hydrodynamic Calculation Method

#### 2.5.1. Wind-Induced Shear Stress

Due to its large water surface area and shallow depth, sediment resuspension in Lake Chaohu is primarily caused by wind-induced disturbances. This is mainly attributed to the fact that wind action generates shear stress on the surface sediment, resulting in its resuspension into the overlying water column. The formula for calculating this shear stress can be considered as the interfacial shear stress between mud and water generated during the propagation of wind waves. Key factors taken into account include wind speed, water depth, wave height, wavelength, period, and the topographical characteristics of the lake. Below is a general equation [29].

(1) $$\tau_{c}=0.5\rho \times f_{c}\times {\left[\pi H/Tsinh(2\pi Z/L)\right]}^{2}$$

*τ*_c_ is the shear stress(N/m^2^); *ρ* is the density of water (g/cm^3^), which is typically taken as 1 for simplicity in calculations; *Z* the depth of water (m); and *f*_c_ is the friction coefficient, and the empirical equation for its calculation [30] is as follows:

(2) $$f_{c}=\left\{\begin{array}{c} \exp\left(-6.0+5.2{\left(H/6\sinh\left(2\pi Z/L\right)d_{50}\right)}^{-0.19}\right),\;H/6\sinh\left(2\pi Z/L\right)d_{50}>1.59\\ \;0.30\;,\;H/6\sinh\left(2\pi Z/L\right)d_{50}\leq 1.59 \end{array}\right.$$

where *d*_50_ is the median particle size of sediment (µm); *H*, *T*, and *L* are the characteristic indices of the traveling wind wave, involving wave height (m), period (s), and wavelength (m), which are generally calculated using the following equation [31]:

(3) $$\left\{\begin{array}{c} gH/U^{2}=0.283tanh\left\{0.530{\left(gZ/U^{2}\right)}^{0.75}\right\}tanh\left\{\frac{0.0125{\left(gF/U^{2}\right)}^{0.42}}{tanh\left[(0.530{\left(gZ/U^{2}\right)}^{0.75}\right]}\right\}\\ gT/2\pi U=1.2tanh\left[0.833{\left(gZ/U^{2}\right)}^{0.375}\right]tanh\left\{\frac{0.077{\left(gF/U^{2}\right)}^{0.25}}{tanh\left[0.833{\left(gZ/U^{2}\right)}^{0.375}\right]}\right\}\\ \;L=gT^{2}tanh(2\pi Z/L)/2\pi \; \end{array}\right.$$

where *U* is the statistical wind speed (m/s) of Chaohu Lake and *F* is the wind area length of the wind field. In this study, it is determined through the statistical meteorological wind speed data, *F* = 17,610 m.

#### 2.5.2. Released Concentration

Based on the detected trace metal contents in the surface sediment (within 5 cm) of the three regions of Lake Chaohu (Appendix A Table A2), along with the detected concentrations of trace metals *N_i_* (mg/kg), sediment resuspension depth *T*_ss_ (mm), surface sediment moisture content *W* (%), and density *ρ* (kg/m^3^), the amount of trace metals released into the water per unit area *M_i_* (mg/m^2^) for each region can be calculated as follows:

(4) $$M_{i}=N_{i}*\left[\left(1-W\right)\rho *\left(1*T_{ss}\right)\right]$$

This study believes that trace metals will be uniformly distributed in water after enough time. Therefore, the average amount of trace metals released into the water across the entire lake area $\bar{M_{i}}$ can be calculated. By combining this with the water surface area *A* (km^2^) and volume *V* (10^8^ m^3^) under different scenarios, the average concentrations of trace metals released into the water $\bar{C_{i}}$ (ug/L) can be determined.

(5) $$\bar{C_{i}}=\bar{M_{i}}*A/V$$

### 2.6. Calculating Ecological Risk Index HC5, RQ, and mixPAF

#### 2.6.1. HC5

In this study, the concentration corresponding to the 5% cumulative frequency on the SSD curve, known as HC5, is conventionally used as an ecological risk assessment indicator [32]. HC5 represents the concentration of a trace metal when 5% of the species are affected. It represents the ecological risk of the trace metal itself, similar to the toxicity factor in the potential ecological risk. The lower the HC5 value is, the higher the ecological risk of the trace metal.

(6) HC(q)=F(x)

where *q* = 0.05.

#### 2.6.2. RQ

The ecotoxicological risk assessment method for a single trace metal indicator is generally the risk quotient (*RQ*), which has been widely used in the ecotoxicological risk assessment of trace metals [33]. The calculation method is as follows:

(7) $${RQ}_{i}=C_{i}/{HC5}_{i}$$

where *C_i_* is the concentration of a certain trace metal index released by sediments into the water body and HC5*_i_* is the ecological risk concentration of a certain trace metal index. The *RQ* value is an ecotoxicological risk value. *RQ* < 1 indicates a negligible ecotoxicological risk, while *RQ* ≥ 1 indicates a potential ecotoxicological risk that should not be excluded.

#### 2.6.3. mixP

To comprehensively assess the combined ecological risk of multiple trace metals, the mix*P* percentage [34] is often used, which evaluates the percentage of species affected by multiple pollutants at a given environmental concentration. Using the previously fitted SSD curves for species, the percentage of species affected by a certain concentration of a trace metal *P_i_* can be obtained (Appendix A Table A3). The combined ecological risk mix*P* can then be calculated using the following equation:

(8) $$mixP=1-\prod_{i=1}^{n}\left(1-P_{i}\right)$$

mix*P* is a probability value representing ecological risk; a higher value indicates a higher combined ecological risk from trace metals in sediments.

## 3. Results

### 3.1. Estimation of Sediment Resuspension Concentration

#### 3.1.1. Sediment Resuspension Depth

Based on the wind speeds *U* in the four scenarios outlined in Appendix A Table A1 and the average water depth *Z* calculated from the lake volume and surface area, the wave height *H*, period *T*, and wavelength *L* can be determined using Equation (3). Using the median grain size of sediment in the western, central, and eastern regions as described in Section 2.2 and applying Equation (2), the friction coefficient *f*_*c*_ for each of the three regions can be calculated. Finally, by substituting the calculated parameters into Equation (1), the shear stress *τ*_c_ for the three regions of Lake Chaohu can be obtained (Appendix A Table A4).

**Figure 3:**
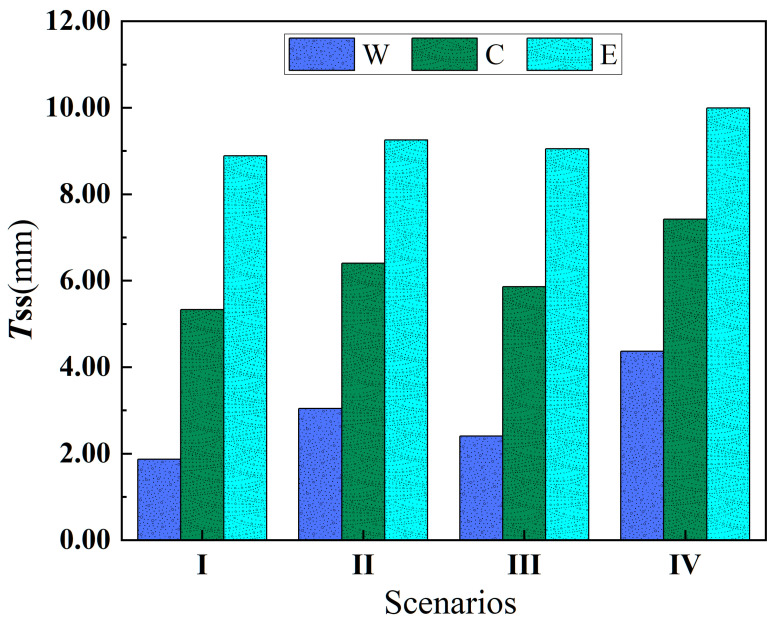
Sediment suspension depth (*T_SS_*) in different regions under four scenarios.

In addition, Luo et al. [35] observed the resuscitation process of representative sediments in the three lake regions under hydraulic disturbance in a cylinder in the laboratory and obtained the corresponding relationship between shear stress and sediment resuscitation depth (*T_SS_*). According to this correspondence, *T_SS_* in the three regions and four situations can be obtained as well (Figure 3).

Figure 3 shows that the sediment *T_SS_* is highest in scenario IV, followed by scenario II, then scenario III, and it is lowest in scenario I. Additionally, in each scenario, the sediment *T_SS_* in the eastern region is larger than that in the central region, and sediment *T_SS_* is the lowest in the western region. The primary reason for this is that the average water depth in a shallow lake fluctuates little throughout the year, making wind action the major factor influencing sediment resuspension. The stronger the wind and waves, the greater the shear stress acting on the sediment, leading to a deeper resuspension depth.

#### 3.1.2. Released Concentrations of Trace Metals

**Figure 4:**
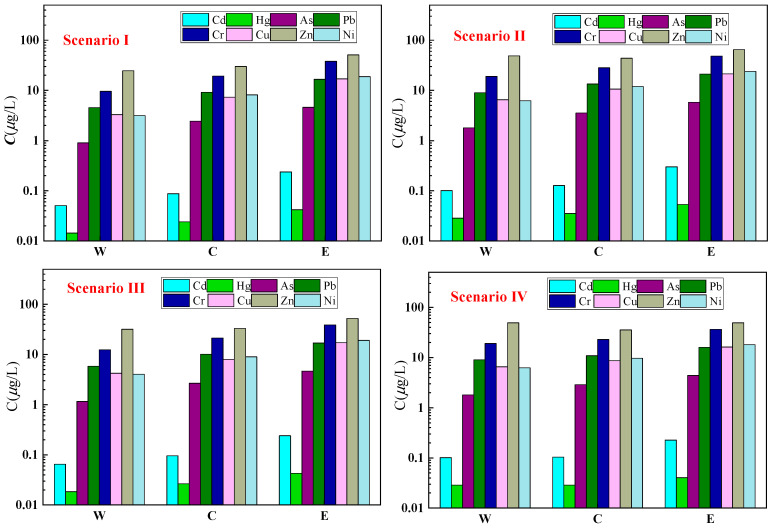
Concentration of trace metals released (*C*) in different regions under four scenarios.

Finally, based on the proportion of trace metals released into the water column from each region *M_i_* and using the calculation methods from Equations (4) and (5), along with the relevant basic data, the concentration of trace metals released into the water for each scenario *C*_i_ can be calculated (Figure 4).

Figure 4 illustrates the distribution of trace metal concentrations *C* released into the water column from the three regions of Chaohu under four different scenarios. It is evident that in scenario II, the concentrations of trace metals released into the water are the highest across all three regions, following the pattern of western region < central region < eastern region. Considering the specific trace metal indices, the differences between regions under each scenario are not easily discernible from the graph due to the varying magnitudes of each indicator’s concentration, necessitating further analysis.

### 3.2. Estimation of Toxicity Threshold of Trace Metals

Using the collected toxicological data for all relevant species (Appendix A Table A5), SSD curves for all species were constructed. The SSD curves for the eight trace metals are shown in Figure 4. According to Equation (6), the HC5 value of trace metals in Chaohu Lake can be obtained. Moreover, the lower the HC5 value, the higher the ecological risk of the trace metal.

**Figure 5:**
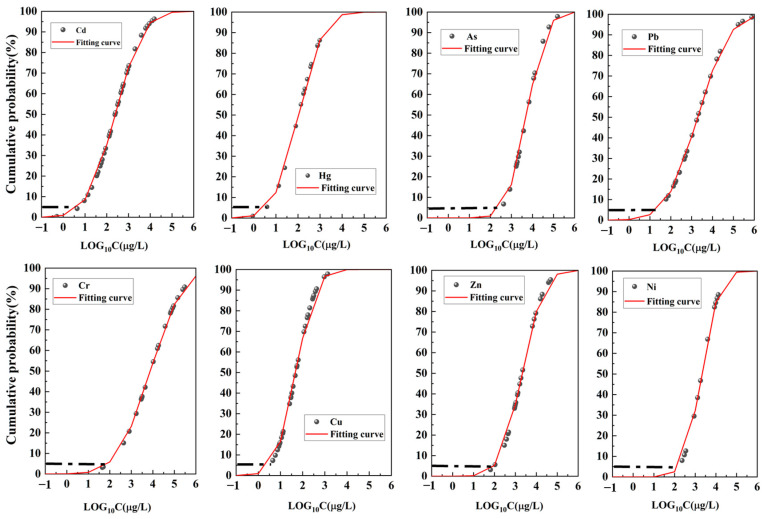
SSD curves for trace metals.

From the SSD curves for the eight trace metals shown in Figure 5, the HC5 values for all relevant species in the water can be determined. The calculated HC5 values for Cd, Hg, As, Pb, Cr, Cu, Zn, and Ni are 5.25, 3.70, 330.58, 21.58, 80.87, 3.33, 96.12, and 161.51 μg/L, respectively. The results indicate that the toxicity to all species, from highest to lowest, is in the order of Cu, Hg, Cd, Pb, Cr, Zn, Ni, and As. These findings are consistent with the HC5 values and toxicity patterns of trace metals in the Yangtze–Huaihe River Basin [15,16].

## 4. Discussion

### 4.1. Risk Assessment of a Single Trace Metal

According to the concentration of sediment released into the water body at 32 monitoring points under four scenarios, the *RQ* values of eight trace metals can be calculated by Equation (7) (Figure 5).

**Figure 6:**
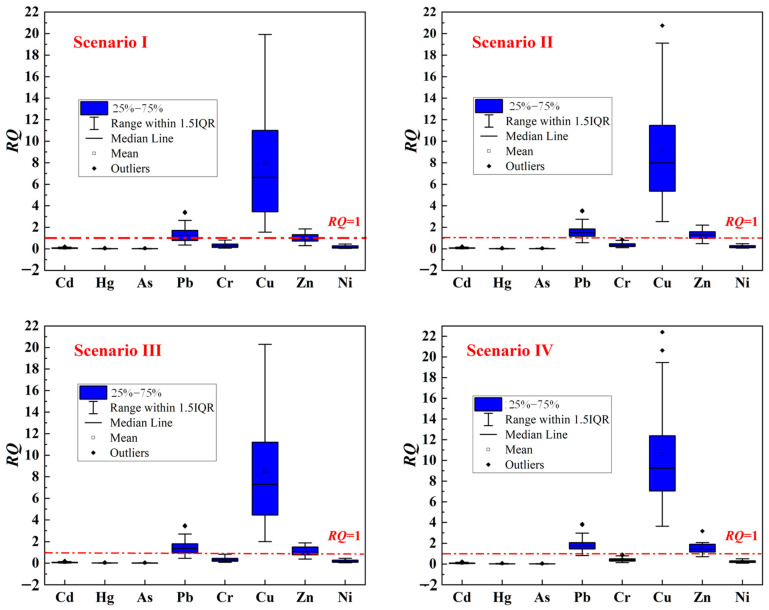
Box plot of *RQ* values of trace metals in lake sediments (IQR: interquartile range).

Figure 6 clearly shows that under all four scenarios, Cu and Pb in the sediments pose a significant potential ecological toxicological risk that cannot be ignored. Zn also presents a notable potential ecological toxicological risk in three scenarios, excluding scenario one. The potential ecological toxicological risks of the other trace metals—Cd, Hg, As, Cr, and Ni—are negligible. Additionally, the *RQ* values indicate that the potential ecological toxicological risk of individual trace metals in the sediments follows the order of Cu > Pb > Zn. These three trace metals are primarily derived from human activities, e.g., industrial and agricultural activities [36,37]. This confirms that the pollution of sediments is mainly due to large-scale industrial and agricultural activities around the lake since the 1970s.

### 4.2. Species-Oriented Ecological Risk Differentiation

**Figure 7:**
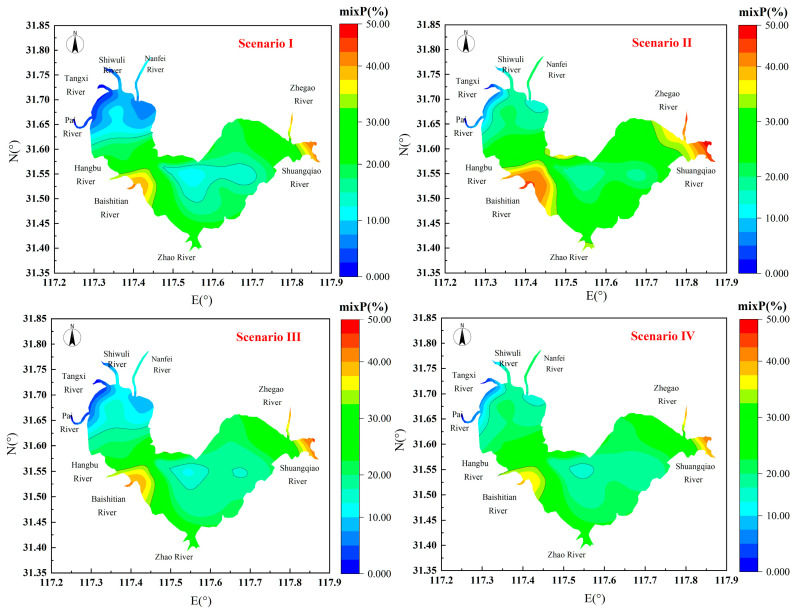
Combined ecological risks of trace metals in sediments to all species.

Using Equation (8), the combined ecological risk for the 32 sampling points in Lake Chaohu under four different scenarios was calculated, and a spatial interpolation was performed to show of the spatial ecological risk contribution rate for the entire lake (Figure 7).

**Figure 8:**
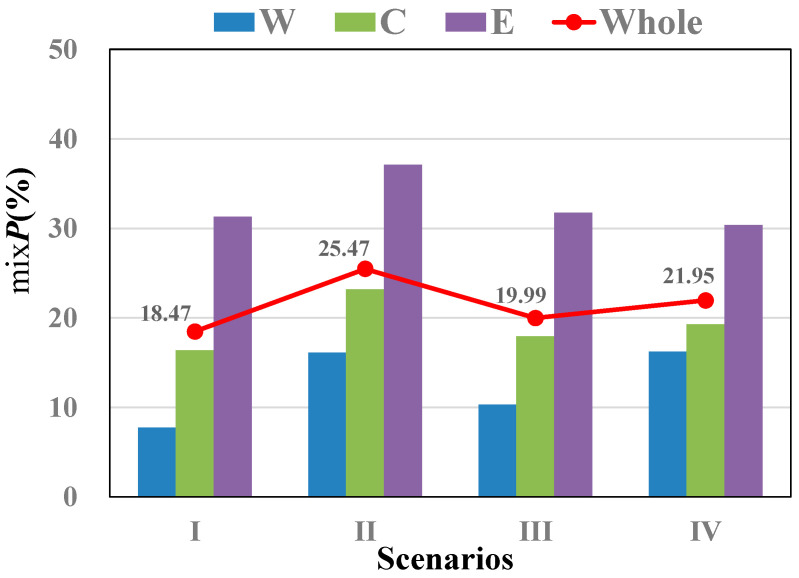
Combined ecological risks of trace metals in sediments under four scenarios.

Figure 7 shows that the mean mix *P* for the eight trace metals in the surface sediments does not exceed 50%, with majority of values ranging between 10% and 30%. Spatially, the overall ecological risk points are generally low, except for two relatively higher-risk areas, which are the Baishitian River inflow region in the central lake and the outlet in the eastern lake. These areas are likely more polluted due to intensive industrial and agricultural activities. For a more intuitive comparison, we have summarized the combined ecological risk values for the three regions and the whole lake (Figure 8).

Figure 8 reveals that the highest calculated ecological toxicological risk for the entire lake is 25.47%, which is close to 24.8%, which was reported by Fang et al. [16], indicating the reliability of our results. Furthermore, the spatial distribution of the sediment’s combined ecological toxicological risk follows the pattern of western region < central region < eastern region. Seasonally, the distribution of the combined ecological toxicological risk in sediments is scenario I (May) < scenario III (September) < scenario IV (November to April of the following year) < scenario II (June to August). The primary reason for this pattern is that during the summer months, heavy rainfall and higher temperatures make it easier for trace metals to be released from the sediments into the overlying water.

### 4.3. Recommendations Based on Ecological Risks of Trace Metals

Based on the assessment of the comprehensive ecotoxicological risk of trace metals in sediments, as well as the spatial and seasonal distribution and risk characteristics of Cu, Pb, and Zn, controlling the ecological risk of sediment trace metals requires strengthening industrial and agricultural pollution source control [38] and implementing phased regional governance [39]. However, it is evident that these measures rely on monitoring the trace metal content in sediments and the results of ecological risk assessments. Therefore, a key method for monitoring and managing lake sediment pollution involves the real-time monitoring of trace metal content in sediments and their trends, assessing ecological risks, establishing a trace metal pollution warning system, and promptly releasing pollution information to guide industrial and agricultural production activities [40].

Furthermore, studies have found that surface sediments in lakes are in an unstable state, alternately undergoing static, resuspension, and settling processes [41]. Thus, for sediments with the dynamic characteristics of trace metal contents, it is essential to evaluate both the potential ecological risks and ecological toxicological risks based on the accumulation and release of trace metals. Combining these two risks will provide the most persuasive trace metal ecological risk predictions.

To achieve a comprehensive assessment of annual ecological risks posed by trace metals in sediments, we propose a pathway by statistically analyzing annual wind speed and frequency and understanding the relationship between wind speed and the threshold shear stress required to resuspend sediments. Then, we can estimate the frequency of sediments being in static versus dynamic states. Combining the potential ecological risk assessments based on sediment trace metal accumulation under static conditions with the ecological toxicological risk assessments based on the release amounts of trace metals from sediments, we can derive an integrated annual ecological risk assessment for sediment trace metals. This approach ensures a detailed and dynamic understanding of sediment behavior and its associated risks, incorporating both static accumulation and resuspension processes.

## 5. Conclusions

This study represents an initial exploration into the ecological risks of trace metals in Chaohu Lake, providing valuable insights into sediment resuspension and its implications. However, we recognize its limitations and consider this work a foundation for more comprehensive future research. Notably, the analysis focuses primarily on directly addressing water and suspended matter dynamics, which are crucial for a more holistic risk assessment. Future studies should incorporate detailed analyses of the potential bioavailability of trace metals, as this plays a critical role in their ecological impact. Bioavailability is influenced by both the chemical and physical composition of sediments (e.g., grain size, organic matter content, clay mineral distribution) and environmental factors such as pH, redox conditions, and ionic strength. Understanding these parameters would provide a more nuanced evaluation of ecological risks and their spatial and temporal variations. By acknowledging these gaps, our study sets the stage for an expanded investigation that integrates sediment, water, and suspended matter analyses. Such a multidisciplinary approach would offer a more robust assessment of environmental risks, ensuring that the conclusions drawn are well-supported and actionable.

## Data Availability

All data generated or analyzed in this study are included in this manuscript. The data that support the findings of this study are available upon request from the corresponding author. The data are not publicly available due to privacy or ethical restrictions.

## Appendix A

**Table A1:** Hydrological and meteorological statistics of Chaohu Lake.

Scenario	Season	Months	Water Level/m	Area/km^2^	Volume/10^8^·m^3^	Wind Speed/m·s^−1^
I	Flood	May	8.50	769.00	21.86	3.37
II		June to August	8.00	764.00	17.85	4.04
III		September	8.50	769.00	21.86	4.07
IV	Non-flood	October to April (Next year)	9.00	775.17	25.81	5.99

**Table A2:** Heavy metal content detected in sediment surface (mg/kg).

Regions	Points	Cd	Hg	As	Pb	Cr	Cu	Zn	Ni
W	W1	0.31	0.163	5.05	40.4	51	25	198	17
	W2	0.63	0.177	8.84	50.1	80	33	209	33
	W3	0.67	0.139	9.04	43.9	73.7	32	227.1	28.1
	W4	0.54	0.097	7.07	24.2	60.6	27	155.2	23.6
	W5	0.32	0.182	11	46.4	171.6	29	215.5	29.7
	W6	0.47	0.169	8.25	43.5	71	30	155	32
	W7	0.35	0.095	4.68	33.9	63	20	214	20
	W8	0.2	0.026	6.52	21.8	67	26	351	22
	W9	0.24	0.011	4.67	20.5	46	15	88	17
	W10	0.16	0.041	3.69	22.5	52	14	79	18
	W11	0.35	0.072	9.8	46	74	31	142.2	39.1
C	C1	0.33	0.113	10.9	33.4	80.5	27	116.6	38.1
	C2	0.35	0.069	9.87	30.7	70.8	25	124.1	28.9
	C3	0.27	0.07	6.57	40.5	62	21	81.5	16.7
	C4	0.39	0.069	8.49	23.8	71.4	28	101.6	31.1
	C5	0.36	0.125	7.25	34.7	67.3	30	153.1	24.5
	C6	0.33	0.152	6.58	29.4	63.4	24	82.1	25.7
	C7	0.2	0.023	7.26	27.3	60.8	18	74.7	27.7
	C8	0.26	0.075	9.24	34.1	69	28	104	29
	C9	0.28	0.084	8.3	22.4	63	20	85	29
	C10	0.27	0.084	10	33.1	68	31	100	27
	C11	0.25	0.029	3.4	24.4	40.6	31	67.1	22.27
E	E1	0.5	0.054	3.1	21.1	47.4	32	68.1	24.8
	E2	0.42	0.028	4.11	20.1	49	20	41	25
	E3	0.44	0.055	5.37	24.1	51.1	27	68.9	34.6
	E4	0.26	0.177	6.58	27.5	60.7	20	92.9	28.2
	E5	0.28	0.062	6.27	32.7	63	20	102	27
	E6	0.37	0.086	11.4	42.3	75	35	94	42
	E7	0.26	0.051	11.4	22.6	74	33	87	32
	E8	0.36	0.064	8.74	20.5	76	22	95	32
	E9	0.46	0.107	11.2	21.1	71	24	100	31
	E10	0.64	0.039	10.3	41.4	73	38	101	38

Remark: “W”, “C”, and “E” represent the three lake regions (western, central, and eastern).

**Table A3:** (**a**) *P*_i_ (%) of each monitoring point in scenario I. (**b**) *P*_i_ (%) of each monitoring point in scenario II. (**c**) *P*_i_ (%) of each monitoring point in scenario III. (**d**) *P*_i_ (%) of each monitoring point in scenario IV.

**(a)**									
**Regions**	**Points**	**Cd**	**Hg**	**As**	**Pb**	**Cr**	**Cu**	**Zn**	**Ni**
W	W1	0.009	0.001	0.000	1.534	0.541	4.867	0.942	0.000
	W2	0.030	0.002	0.000	1.865	0.853	6.832	1.018	0.003
	W3	0.033	0.001	0.000	1.655	0.787	6.588	1.145	0.002
	W4	0.024	0.000	0.000	0.941	0.646	5.360	0.659	0.001
	W5	0.010	0.002	0.000	1.740	1.745	5.852	1.063	0.002
	W6	0.019	0.002	0.000	1.641	0.758	6.098	0.658	0.003
	W7	0.012	0.000	0.000	1.302	0.672	3.640	1.053	0.001
	W8	0.004	0.000	0.000	0.849	0.715	5.113	2.057	0.001
	W9	0.006	0.000	0.000	0.798	0.486	2.443	0.270	0.000
	W10	0.003	0.000	0.000	0.876	0.552	2.212	0.225	0.001
	W11	0.041	0.001	0.000	3.361	1.644	14.601	1.738	0.036
C	C1	0.037	0.003	0.000	2.575	1.773	12.755	1.330	0.034
	C2	0.041	0.001	0.000	2.395	1.580	11.800	1.448	0.017
	C3	0.027	0.001	0.000	3.027	1.399	9.827	0.800	0.004
	C4	0.048	0.001	0.000	1.916	1.592	13.224	1.098	0.021
	C5	0.042	0.004	0.000	2.660	1.509	14.147	1.915	0.011
	C6	0.037	0.006	0.000	2.307	1.429	11.315	0.808	0.013
	C7	0.017	0.000	0.000	2.163	1.374	8.294	0.703	0.015
	C8	0.025	0.001	0.000	2.620	1.543	13.224	1.135	0.017
	C9	0.029	0.002	0.000	1.815	1.420	9.321	0.850	0.017
	C10	0.027	0.002	0.000	2.555	1.523	14.601	1.074	0.015
	C11	0.079	0.001	0.000	3.766	1.921	28.077	1.794	0.059
E	E1	0.210	0.004	0.000	3.350	2.199	28.734	1.829	0.075
	E2	0.165	0.001	0.000	3.219	2.263	19.819	0.909	0.076
	E3	0.176	0.004	0.001	3.729	2.346	25.306	1.858	0.154
	E4	0.084	0.037	0.001	4.140	2.713	19.819	2.717	0.099
	E5	0.093	0.005	0.001	4.733	2.798	19.819	3.046	0.090
	E6	0.139	0.010	0.004	5.739	3.228	30.626	2.757	0.230
	E7	0.084	0.003	0.004	3.541	3.193	29.377	2.504	0.130
	E8	0.133	0.005	0.002	3.272	3.263	21.479	2.793	0.130
	E9	0.188	0.014	0.004	3.350	3.087	23.062	2.973	0.122
	E10	0.292	0.002	0.003	5.649	3.158	32.410	3.010	0.187
**(b)**									
**Regions**	**Points**	**Cd**	**Hg**	**As**	**Pb**	**Cr**	**Cu**	**Zn**	**Ni**
W	W1	0.029	0.006	0.000	2.801	1.070	10.740	2.371	0.003
	W2	0.086	0.007	0.000	3.347	1.625	14.209	2.537	0.019
	W3	0.094	0.004	0.000	3.002	1.509	13.791	2.812	0.013
	W4	0.068	0.002	0.000	1.793	1.259	11.634	1.731	0.008
	W5	0.031	0.008	0.000	3.143	3.129	12.510	2.636	0.015
	W6	0.056	0.007	0.000	2.979	1.458	12.942	1.728	0.018
	W7	0.035	0.002	0.000	2.413	1.306	8.431	2.613	0.005
	W8	0.014	0.000	0.000	1.631	1.382	11.189	4.686	0.007
	W9	0.019	0.000	0.000	1.541	0.969	6.029	0.783	0.003
	W10	0.010	0.000	0.000	1.678	1.090	5.542	0.667	0.004
	W11	0.072	0.003	0.001	4.541	2.296	20.557	2.820	0.085
C	C1	0.066	0.007	0.001	3.528	2.467	18.241	2.200	0.081
	C2	0.072	0.003	0.001	3.295	2.210	17.024	2.381	0.043
	C3	0.049	0.003	0.000	4.113	1.969	14.465	1.370	0.011
	C4	0.085	0.003	0.001	2.666	2.227	18.834	1.841	0.051
	C5	0.075	0.009	0.000	3.638	2.116	19.992	3.086	0.029
	C6	0.066	0.013	0.000	3.180	2.008	16.401	1.384	0.033
	C7	0.031	0.000	0.000	2.991	1.936	12.430	1.215	0.039
	C8	0.046	0.003	0.001	3.588	2.162	18.834	1.898	0.043
	C9	0.052	0.004	0.001	2.532	1.997	13.798	1.451	0.043
	C10	0.049	0.004	0.001	3.502	2.135	20.557	1.803	0.037
	C11	0.111	0.002	0.000	4.534	2.359	33.139	2.433	0.099
E	E1	0.288	0.006	0.000	4.049	2.689	33.845	2.478	0.125
	E2	0.229	0.002	0.001	3.896	2.764	24.084	1.270	0.128
	E3	0.243	0.006	0.001	4.491	2.862	30.139	2.515	0.250
	E4	0.118	0.056	0.002	4.967	3.294	24.084	3.613	0.165
	E5	0.131	0.008	0.002	5.653	3.394	24.084	4.028	0.150
	E6	0.192	0.015	0.007	6.806	3.897	35.868	3.663	0.367
	E7	0.118	0.005	0.007	4.273	3.857	34.535	3.343	0.214
	E8	0.185	0.008	0.004	3.958	3.938	25.932	3.709	0.214
	E9	0.258	0.023	0.007	4.049	3.733	27.682	3.937	0.200
	E10	0.396	0.003	0.006	6.704	3.816	37.760	3.983	0.302
**(c)**									
**Regions**	**Points**	**Cd**	**Hg**	**As**	**Pb**	**Cr**	**Cu**	**Zn**	**Ni**
W	W1	0.015	0.003	0.000	1.929	0.701	6.636	1.345	0.001
	W2	0.045	0.003	0.000	2.330	1.090	9.111	1.448	0.006
	W3	0.050	0.002	0.000	2.076	1.007	8.807	1.619	0.004
	W4	0.035	0.001	0.000	1.203	0.833	7.263	0.955	0.003
	W5	0.015	0.003	0.000	2.180	2.180	7.885	1.509	0.005
	W6	0.029	0.003	0.000	2.060	0.972	8.194	0.954	0.006
	W7	0.018	0.001	0.000	1.647	0.865	5.054	1.495	0.002
	W8	0.007	0.000	0.000	1.088	0.919	6.950	2.828	0.002
	W9	0.009	0.000	0.000	1.025	0.632	3.474	0.406	0.001
	W10	0.005	0.000	0.000	1.122	0.715	3.163	0.342	0.001
	W11	0.047	0.002	0.000	3.629	1.790	15.965	1.968	0.045
C	C1	0.043	0.004	0.001	2.790	1.928	14.002	1.513	0.042
	C2	0.047	0.001	0.000	2.598	1.721	12.983	1.645	0.022
	C3	0.031	0.002	0.000	3.273	1.526	10.867	0.918	0.005
	C4	0.055	0.001	0.000	2.084	1.734	14.502	1.253	0.026
	C5	0.049	0.005	0.000	2.880	1.644	15.484	2.165	0.014
	C6	0.043	0.008	0.000	2.503	1.558	12.463	0.928	0.016
	C7	0.019	0.000	0.000	2.349	1.499	9.213	0.809	0.020
	C8	0.030	0.002	0.000	2.839	1.681	14.502	1.295	0.022
	C9	0.033	0.002	0.000	1.975	1.549	10.322	0.975	0.022
	C10	0.031	0.002	0.001	2.768	1.659	15.965	1.226	0.018
	C11	0.081	0.001	0.000	3.821	1.952	28.453	1.838	0.061
E	E1	0.215	0.004	0.000	3.400	2.234	29.114	1.874	0.078
	E2	0.170	0.001	0.000	3.268	2.298	20.130	0.933	0.079
	E3	0.181	0.004	0.001	3.784	2.382	25.662	1.902	0.160
	E4	0.086	0.038	0.001	4.199	2.754	20.130	2.779	0.103
	E5	0.096	0.005	0.001	4.799	2.840	20.130	3.114	0.094
	E6	0.142	0.010	0.004	5.816	3.276	31.017	2.819	0.238
	E7	0.086	0.003	0.004	3.594	3.240	29.761	2.562	0.136
	E8	0.137	0.005	0.002	3.321	3.311	21.805	2.856	0.136
	E9	0.192	0.015	0.004	3.400	3.133	23.401	3.040	0.127
	E10	0.299	0.002	0.003	5.725	3.205	32.810	3.077	0.194
**(d)**									
**Regions**	**Points**	**Cd**	**Hg**	**As**	**Pb**	**Cr**	**Cu**	**Zn**	**Ni**
W	W1	0.029	0.006	0.000	2.817	1.077	10.817	2.391	0.003
	W2	0.087	0.007	0.000	3.365	1.635	14.302	2.559	0.019
	W3	0.095	0.004	0.000	3.019	1.518	13.882	2.836	0.013
	W4	0.069	0.002	0.000	1.804	1.267	11.715	1.746	0.008
	W5	0.031	0.008	0.000	3.160	3.146	12.595	2.658	0.015
	W6	0.056	0.007	0.000	2.996	1.467	13.029	1.743	0.018
	W7	0.036	0.002	0.000	2.427	1.314	8.494	2.635	0.005
	W8	0.014	0.000	0.000	1.641	1.391	11.268	4.721	0.007
	W9	0.020	0.000	0.000	1.551	0.975	6.079	0.791	0.003
	W10	0.010	0.000	0.000	1.689	1.097	5.588	0.674	0.004
	W11	0.053	0.002	0.001	3.863	1.918	17.153	2.177	0.054
C	C1	0.048	0.005	0.001	2.978	2.065	15.093	1.680	0.050
	C2	0.053	0.002	0.001	2.775	1.845	14.019	1.825	0.026
	C3	0.036	0.002	0.000	3.488	1.638	11.783	1.027	0.007
	C4	0.062	0.002	0.000	2.232	1.859	15.618	1.396	0.031
	C5	0.055	0.006	0.000	3.074	1.764	16.648	2.391	0.018
	C6	0.048	0.009	0.000	2.676	1.672	13.472	1.037	0.020
	C7	0.022	0.000	0.000	2.512	1.610	10.027	0.906	0.024
	C8	0.034	0.002	0.001	3.030	1.803	15.618	1.441	0.027
	C9	0.038	0.003	0.000	2.116	1.662	11.205	1.090	0.027
	C10	0.036	0.003	0.001	2.956	1.780	17.153	1.366	0.022
	C11	0.074	0.001	0.000	3.645	1.854	27.246	1.700	0.053
E	E1	0.199	0.003	0.000	3.240	2.123	27.894	1.734	0.068
	E2	0.156	0.001	0.000	3.113	2.185	19.135	0.857	0.069
	E3	0.167	0.004	0.001	3.609	2.265	24.518	1.761	0.141
	E4	0.079	0.034	0.001	4.009	2.622	19.135	2.583	0.091
	E5	0.088	0.005	0.001	4.588	2.705	19.135	2.899	0.082
	E6	0.131	0.009	0.004	5.570	3.123	29.760	2.621	0.211
	E7	0.079	0.003	0.004	3.427	3.089	28.528	2.380	0.119
	E8	0.126	0.005	0.002	3.164	3.157	20.761	2.656	0.119
	E9	0.177	0.013	0.004	3.240	2.986	22.314	2.829	0.112
	E10	0.277	0.002	0.003	5.482	3.055	31.522	2.864	0.172

**Table A4:** Shear stress of sediments under wave action.

Scenarios	Wind Speed /m·s^−1^	Wave Characteristic Value			*τ_c_*/N·m^−2^		
		*H*/m	*T*/s	*L*/m	Western	Central	Eastern
I	3.37	0.18	1.66	4.28	0.021	0.033	0.028
II	4.04	0.22	1.80	5.02	0.092	0.130	0.118
III	4.07	0.23	1.84	5.27	0.053	0.077	0.072
IV	5.99	0.35	2.29	8.06	0.173	0.229	0.224

**Table A5:** Species mean acute values (SMAVs) for trace metals to construct species sensitivity distribution (SSD) curves (μg/L).

**Species**	**Cu**	**Species**	**Pb**	**Species**	**Zn**	**Species**	**Cr**
Ceriodaphniapulchella	4.52	Daphnia similis	60	Ceriodaphniadubia	65	Ceriodaphniareticulata	45
Daphnia longispina	4.56	Tubifextubifex	77	Daphnia pulex	107	Daphnia pulex	48
Moinamacrocopa	5.9	Daphnia magna	135	Ceriodaphniareticulata	300	Simocephalusvetulus	50
Ceriodaphniadubia	7.47	Ceriodaphniadubia	162	Daphnia longispina	375	Daphnia magna	438
Daphnia galeata	8.5	Gammaruspulex	175	Cyprinuscarpio	450	Chironomusplumosus	800
Ceriodaphniareticulata	8.55	Caenorhabditiselegans	260	Simocephalusvetulus	473	Daphnia ambigua	1700
Bosminalongirostris	9.2	Cyprinuscarpio	262	Simocephalusexspinosus	911	Brachionuscalyciflorus	2900
Chydorusovalis	9.6	Daphnia carinata	444	Daphnia magna	952	Tubifextubifex	3032
Simocephalusexspinosus	11.3	Chironomusplumosus	500	Daphnia galeata	1001	Gammarus sp.	3200
Daphnia pulex	12.8	Daphnia hyalina	600	Misgurnusanguillicaudatus	1050	Bellamyaquadrata	4412
Simocephalusvetulus	13.4	Ceriodaphniareticulata	1050	Ceriodaphniapulchella	1263	Cyclops sp.	10,470
Brachionuscalyciflorus	26	Daphnia pulex	1745	Chydorussphaerius	1326	Carassiusauratus	16,490
Gammaruspulex	29.6	Chironomustentans	2178	Chydorusovalis	1627	Pelteobagrusfulvidraco	18,317
Acanthocyclopsviridis	30	Moinamacrocopa	3133	Bellamyaquadrata	1863	Ranachensinensis	37,511
Daphnia magna	32.6	Simocephalusvetulus	4500	Gammaruslacustris	2240	Daphnia galeata	65,600
Daphnia carinata	37.3	Lumbriculusvariegatus	8000	Ctenopharyngodonidellus	6494	Ctenopharyngodonidellus	71,190
Chydorussphaerius	46.3	Rhodeussinensis	16,174	Eriocheirsinensis	7908	Monopterusalbus	73,083
Xenocypris sp.	55	Limnodrilushoffmeisteri	23,441	Chironomusplumosus	9500	Daphnia carinata	83,150
Tubifextubifex	56.5	Eriocheirsinensis	169,262	Pseudorasboraparva	15,794	Cyclops viridis	92,500
Bellamyaquadrata	63.3	Carassiusauratus	277,546	Rhodeusocellatus	19,225	Sinipercachuatsi	144,000
Misgurnusmizolepis	115	Procambarusclarkii	751,570	Carassiusauratus	38,356	Hypophthalmichthys molitrix	248,000
Hypophthalmichthys molitrix	131			Cipangopaludinachinensis	42,173	Pseudorasboraparva	301,350
Ctenopharyngodonidellus	162			Ranalimnocharis	48,698		
Cyprinuscarpio	174						
Gammaruslacustris	212						
Megalobramapellegrini	282						
Chironomusplumosus	300						
Lumbriculusvariegatus	320						
Carassiusauratus	380						
Chironomustentans	426						
Diaphanosomabrachyurum	950						
Procambarusclarkii	1350						
**Species**	**Cd**	**Species**	**As**	**Species**	**Hg**	**Species**	**Ni**
Acanthocyclopsviridis	0.5	Hyalellaazteca	426	Daphnia carinata	0.9	Raphidocelissubcapitata	233
Moinairrasa	4.34	Bosminalongirostris	850	Daphnia pulex	4	Chironomusplumosus	300
Simocephalusserrulatus	9.26	Simocephalusvetulus	1700	Daphnia magna	13.5	Desmodesmussubspicatus	350
Daphnia magna	13.7	Ceriodaphniareticulata	1800	Pelteobagrusvachelli	25.6	Hyalellaazteca	890
Macrobranchiumnipponense	20.5	Daphnia pulex	1900	Amnicola sp.	80	Cyprinuscarpio	1300
Daphnia galeata	34.6	Daphnia carinata	2157	Megalobramapellegrini	137	Daphnia magna	1800
Brachionuscalyciflorus	36.6	Ceriodaphniadubia	2400	Bellamyaquadrata	180	Brachionuscalyciflorus	4000
Gammaruslacustris	40.1	Daphnia magna	3800	Cyprinuscarpio	183	Chironomus sp.	8600
Daphnia pulex	49.9	Chironomus sp.	6900	Rhodeussinensis	204	Carassiusauratus	9820
Daphnia hyalina	55	Philodinaroseola	11,509	Pseudorasboraparva	264	Amnicola sp.	11,400
Ceriodaphniadubia	56.3	Cloeondipterum	13,000	Ctenopharyngodonidellus	375	Gammarus sp.	13,000
Moinamacrocopa	62.5	Carassiusauratus	32,000	Rhodeusocellatus	407		
Moinadubia	77	Scenedesmusquadricauda	61,000	Misgurnusanguillicaudatus	773		
Simocephalusvetulus	89.3	Tubifextubifex	155,779	Chironomusplumosus	797		
Gammaruspulex	89.4			Chironomustentans	971		
Ceriodaphniareticulata	129						
**Species**	**Cd**	**Species**	**Cd**				
Daphnia carinata	138	Limnodrilushoffmeisteri	3919				
Chydorussphaerius	149	Eriocheirsinensis	3928				
Branchiurasowerbyi	240	Megalobramapellegrini	6188				
Cyprinuscarpio	254	Rhodeussinensis	7284				
Tubifextubifex	320	Carassiusauratus	9124				
Chironomusplumosus	346	Chironomustentans	12,394				
Spirospermanikolskyi	450	Ctenopharyngodonidellus	15,540				
Glossiphoniacomplanata	480						
Alonaaffinis	546						
Daphnia obtusa	580						
Xenocypris sp.	830						
Cloeondipterum	926						
Procambarusclarkii	1040						
Diaphanosomabrachyurum	1060						
Caenorhabditiselegans	2000						

## References

[1] Murava R.T., Norgbey E., Zhu X. (2024). Spatial Distribution, Risk Index, and Correlation of Trace metals in the Chuhe River (Yangtze Tributary): Preliminary Research Analysis of Surface Water and Sediment Contamination. Appl. Sci..

[2] Mitra A., Roy M., Pal S. (2023). Ecological Risk Assessment of Trace metals in the Coastal Sediment in the South-western Bay of Bengal. Front. Environ. Sci..

[3] Qin Y., Tao Y. (2022). Pollution status of trace metals and metalloids in Chinese lakes: Distribution, bioaccumulation and risk assessment. Ecotoxicological Environ. Saf..

[4] Wuttikul N., Paopongpan S., Phetrungnapha N. (2023). Microplastics and Trace metals in the Sediment of Songkhla Lagoon: Distribution and Risk Assessment. Front. Environ. Sci..

[5] Su K., Wang Q., Li L., Cao R., Xi Y. (2022). Water quality assessment of Lugu Lake based on Nemerow pollution index method. Sci. Rep..

[6] Yang J., Sun F., Su H., Tao Y., Chang H. (2022). Multiple Risk Assessment of Trace metals in Surface Water and Sediment in Taihu Lake, China. Int. J. Environ. Res. Public Health.

[7] Reimann C., de Caritat P. (2017). Establishing geochemical baselines for trace metals. Environ. Geochem. Health.

[8] Iwasaki Y., Takeshita K.M., Ueda K., Naito W. (2023). Estimating species sensitivity distributions for microplastics by quantitatively considering particle characteristics using a recently created ecotoxicity database. Microplastics Nanoplastics.

[9] Ogbeide E., Zhang Y., Li W., Paterson R.A., Jiang G., Miao A. (2018). Application of Species Sensitivity Distributions to Derive Safe Environmental Concentrations for Pollutants: A Comprehensive Review. Sci. Total Environ..

[10] Del Signore A., Hendriks A.J., Lenders H.J., Leuven R.S., Breure A.M. (2016). Development and application of the SSD approach in scientific case studies for ecological risk assessment. Environ. Toxicol. Chem..

[11] Albarano L., Lofrano G., Costantini M., Zupo V., Carraturo F., Guida M., Libralato G. (2021). Comparison of in situ sediment remediation amendments: Risk perspectives from species sensitivity distribution. Environ. Pollut..

[12] Hoppe S., Sundbom M., Borg H., Breitholtz M. (2015). Predictions of Cu toxicity in three aquatic species using bioavailability tools in four Swedish soft freshwaters. Environ. Sci. Eur..

[13] Yang X., Leng M., Ge X., Wu X., Liu H., Lin G., Huang Z., Chen Y. (2024). Characterization and Risk Assessment of Nutrient and Trace metal Pollution in Surface Sediments of Representative Lakes in Yangxin County, China. Sustainability.

[14] Fox D.R., van Dam R.A., Fisher R., Batley G.E., Tillmanns A.R., Thorley J., Schwarz C.J., Spry D.J., McTavish K. (2021). Recent developments in species sensitivity distribution modeling. Environ. Toxicol. Chem..

[15] Zhang S., Zeng X., Sun P., Ni T. (2023). Ecological risk characteristics of sediment-bound trace metals in large shallow lakes for aquatic organisms: The case of Taihu Lake, China. J. Environ. Manag..

[16] Fang T., Yang K., Wang H., Fang H., Liang Y., Zhao X., Gao N., Li J., Lu W., Cui K. (2022). Trace metals in sediment from Chaohu Lake in China: Bioavailability and probabilistic risk assessment. Sci. Total Environ..

[17] Wu J., Yue W., Li Q. (2022). Pollution Assessment and SSD-Based Ecological Assessment of Trace metals in Multimedia in the Coast of Southeast China. Int. J. Environ. Res. Public Health.

[18] Nazeer S., Hashmi M.Z., Malik R.N. (2014). Trace metals distribution, risk assessment and water quality characterization by water quality index of the River Soan, Pakistan. Ecol. Indic..

[19] Luo W., Yue Y., Lu J., Pang L., Zhu S. (2022). Sediment phosphate release flux under hydraulic disturbances in the shallow lake of Chaohu, China. Environ. Sci. Pollut. Res..

[20] Luo W., Pan Y., Fan Y., Lu J., Zhu S. (2024). Distribution Patterns of Sediment Organic Carbon Stocks in Shallow Lakes and the Significance for Sustainable Lake Management: Chaohu Lake in Eastern China as a Case Study. Land.

[21] Liu E., Shen J., Birch G.F., Yang X., Wu Y., Xue B. (2012). Human-induced change in sedimentary trace metals and phosphorus in Chaohu Lake, China, over the past half-millennium. J. Paleolimnol..

[22] Fang T., Lu W., Cui K., Li J., Yang K., Zhao X., Liang Y., Li H. (2019). Distribution, bioaccumulation and trophic transfer of trace metals in the food web of Chaohu Lake, Anhui, China. Chemosphere.

[23] Zhu S., Zhang Z., Zagar D. (2018). Mercury transport and fate models in aquatic systems: A review and synthesis. Sci. Total Environ..

[24] Zhang H., Huo S., Yeager K.M., Xi B., Zhang J., Wu F. (2019). A historical sedimentary record of mercury in a shallow eutrophic lake: Impacts of human activities and climate change. Engineering.

[25] Amato E.D., Wadige C.P.M.M., Taylor A.M., Maher W.A., Simpson S.L., Jolley D.F. (2018). Field and laboratory evaluation of DGT for predicting metal bioaccumulation and toxicity in the freshwater bivalve Hyridella australis exposed to contaminated sediments. Environ. Pollut..

[26] Gu Y., Huang H., Jiang S., Gong X., Liao X., Dai M. (2022). Appraising ecotoxicological risk of mercury species and their mixtures in sediments to aquatic biota using diffusive gradients in thin films (DGT). Sci. Total Environ..

[27] Li X., Yang Y., Yang J., Fan Y., Li H. (2021). Rapid diagnosis of trace metal pollution in lake sediments based on environmental magnetism and machine learning. J. Hazard. Mater..

[28] Wu C., Wu S., Wu X., Dai J., Gao A., Yang F. (2021). Numerical investigation of the effects of aquatic vegetation on wind-induced wave and current characteristics in shallow lakes. Front. Environ. Sci..

[29] Hawley N., Lee C.H. (1999). Sediment resuspension and transport in lake Michigan during unstratified period. Sedimentology.

[30] Lou J., Ridd P.V. (1996). Wave-current bottom shear stresses and sediment resuspension in Cleveland Bay, Australia. Coast. Eng..

[31] Hawley N. (2000). Sediment resuspension near the Keweenaw Peninsula, Lake Superior during the fall and winter. J. Great Lakes Res..

[32] He W., Qin N., Kong X.Z., Liu W.X., He Q.S., Wang Q.M., Yang C., Jiang Y.J., Yang B., Wu W.J. (2014). Water quality benchmarking (WQB) and priority control screening (PCS) of persistent toxic substances (PTSs) in China: Necessity, method and a case study. Sci. Total Environ..

[33] Backhaus T., Faust M. (2012). Predictive environmental risk assessment of chemical mixtures: A conceptual framework. Sci. Total Environ..

[34] Cui Z., Wang Y., Zhao N., Yu R., Xu G., Yu Y. (2018). Spatial distribution and risk assessment of trace metals in paddy soils of Yongshuyu irrigation area from Songhua River Basin, Northeast China. Chin. Geogr. Sci..

[35] Luo W., Lu J., Zhu S., Yue Y., Xiao L. (2022). Investigation of the impact of hydrodynamic conditions on sediment resuspension in shallow lakes. Int. J. Digit. Earth.

[36] Shao D., Zhan Y., Zhou W., Zhu L. (2016). Current status and temporal trend of trace metals in farmland soil of the Yangtze River Delta Region: Field survey and metanalysis. Environ. Pollut..

[37] Liu J., Wu J., Feng W., Li X. (2020). Ecological risk assessment of trace metals in water bodies around typical copper mines in China. Int. J. Environ. Res. Public Health.

[38] Smith J., Wang L., Thompson R. (2015). Characteristics and Ecological Risk Assessment of Trace metal Pollution in Lake Sediments. Environ. Sci. Res..

[39] Johnson P., Liu J., Wang H. (2018). Trace metal Pollution and Risk Assessment in Lake Sediments. J. Lake Sci..

[40] Lee N., Zhang H., Zhao X. (2019). Application of Eco-Islands in the Management of Trace metal Pollution in Lake Sediments. Environ. Sci. Technol..

[41] Lee M.S., Lee J.H., An Y.J., Park C.H., Lee S.H., Park J.H., Lee J.K., Park T.J. (2020). Development of water quality criteria for arsenic to protect aquatic life based on species sensitivity distribution. Ecotoxicol. Environ. Saf..

